# Detection of Road-Surface Anomalies Using a Smartphone Camera and Accelerometer

**DOI:** 10.3390/s21020561

**Published:** 2021-01-14

**Authors:** Taehee Lee, Chanjun Chun, Seung-Ki Ryu

**Affiliations:** Future Infrastructure Research Center, Korea Institute of Civil Engineering and Building Technology (KICT), Goyang 10223, Korea; thlee420@kict.re.kr (T.L.); chanjunchun@kict.re.kr (C.C.)

**Keywords:** smartphone, road-surface damage, three-axis acceleration, dual-acquisition system, artificial intelligence

## Abstract

Road surfaces should be maintained in excellent condition to ensure the safety of motorists. To this end, there exist various road-surface monitoring systems, each of which is known to have specific advantages and disadvantages. In this study, a smartphone-based dual-acquisition method system capable of acquiring images of road-surface anomalies and measuring the acceleration of the vehicle upon their detection was developed to explore the complementarity benefits of the two different methods. A road test was conducted in which 1896 road-surface images and corresponding three-axis acceleration data were acquired. All images were classified based on the presence and type of anomalies, and histograms of the maximum variations in the acceleration in the gravitational direction were comparatively analyzed. When the types of anomalies were not considered, it was difficult to identify their effects using the histograms. The differences among histograms became evident upon consideration of whether the vehicle wheels passed over the anomalies, and when excluding longitudinal anomalies that caused minor changes in acceleration. Although the image-based monitoring system used in this research provided poor performance on its own, the severity of road-surface anomalies was accurately inferred using the specific range of the maximum variation of acceleration in the gravitational direction.

## 1. Introduction

Various types of damage may occur on road surfaces owing to external factors—such as the weather, overloaded vehicles, and traffic volume—and internal factors—such as defects in materials and cross-sections. For example, torrential rain in the summer causes potholes by separating the materials of the asphalt pavement layer, increases in temperature and humidity are the main cause of the blow-up phenomenon, and repeated vehicular loads and overloaded vehicles accelerate plastic deformation and fatigue cracking of the road surface. In addition, when pavement materials are defective, flushing—in which asphalt leaks along the vehicle wheel paths—or rutting—in which the pavement surface is compressed and deformed along the wheel paths—may occur. As a road surface in poor condition may cause serious risks to motorists, systems capable of promptly and accurately managing the road surface have been actively developed [1,2,3,4,5].

Road-surface management begins with the prompt detection of road-surface anomalies. Many studies have been conducted in this regard. Methods used for the detection of road-surface damage can be classified into: (a) identification of vehicular vibration types; (b) measurements using laser irradiation of the road surface; and (c) image recognition [6]. Vibration-based detection methods measure the vibration of a vehicle during motion using an attached three-axis or vertical accelerometer [7,8,9,10,11,12,13,14,15,16,17]. This approach requires several filtering steps to extract only the vibrations caused by the damage to the road surface. Vibration-based detection is suitable for real-time evaluation of road-surface conditions at a low cost. However, it cannot measure road-surface damage in areas other than the vehicle wheel paths and cannot identify the size of the road-surface damage [18].

Laser measurement-based detection methods use special equipment installed on a separate inspection vehicle, such as a laser scanner, to convert the road surface into a three-dimensional (3D) object in a coordinate system [19,20,21,22]. This approach can directly calculate various indicators for the precise evaluation of the road-surface condition. However, real-time processing is difficult at high-driving speeds because of the increased number of calculations required. Furthermore, considerable expense is incurred by the introduction of new machinery and its operation.

Image-recognition-based detection methods can analyze road-surface conditions over a wide area at a reasonable cost. This approach has recently attracted attention owing to the development of image-recognition technology using deep neural networks (DNNs) [23,24,25,26,27,28]. Road-surface damage identification using a DNN can capture images of the road surface in real time as the vehicle travels at a normal driving speed, providing detailed information such as the size of the damaged regions. However, many image datasets with accurate labels of the road-surface conditions are required to train a DNN. Additionally, efforts must be expended to improve the recognition rate by excluding factors that interfere with analysis, such as changes in the road-surface color and illumination under various types of weather, shadows on the road, other traveling vehicles, and road signs.

A combination of different measurement techniques could be expected to compensate for the disadvantages of each road-surface anomaly detection method. Smartphones provide an optimal platform for testing a road-surface anomaly detection method that employs both a DNN inference model to identify road-surface anomalies and acceleration data acquisition because they are equipped with built-in processors, LTE communication modules, three-axis accelerometers, gyroscopes, and cameras. In recent years, both image-recognition- and vibration-based detection methods have been researched using smartphones, but few studies have attempted to combine these two different methods. The development of a smartphone-based road-surface anomaly detection system and associated mobile application could eventually allow information describing roadway hazards to be distributed to users in real time, much like traffic congestion information is currently distributed by navigation applications. Furthermore, a smartphone-based detection system would considerably expand the monitoring capacity of personnel and agencies devoted to road maintenance, allowing repairs to be targeted to the areas with the most urgent need. Finally, the dual-acquisition method demonstrated in this study could be applied in vehicle black boxes to realize widespread implementation.

In this study, the software was accordingly developed to acquire road-surface images from a smartphone camera for use in an image-recognition-based fully convolutional neural network (FCN) model developed in a previous study [28] and acquire acceleration data using the accelerometer built into a smartphone. The developed software was installed in an android-based smartphone (which is Samsung Galaxy s10), and the possibility of combining the two different methods to detect road-surface anomalies was examined based on an analysis of the road-surface images and acceleration measurement results acquired during driving.

## 2. Collection of Road-Surface Anomaly Information Using a Smartphone Camera and Accelerometer

### 2.1. Overall Flow of Data Acquisition

**Figure 1:**
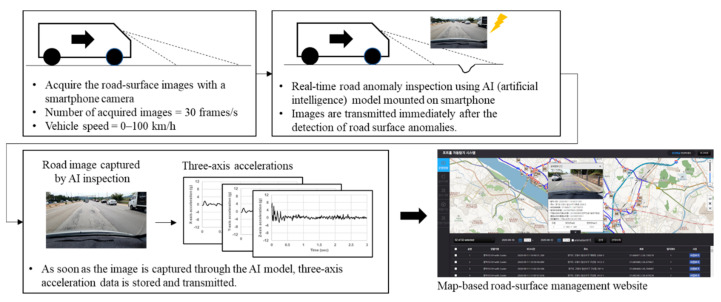
Overall image acquisition flow and three-axis accelerations with a smartphone.

Figure 1 shows the overall flow of the proposed measurement process using the smartphone camera and accelerometer. First, road-surface images with a resolution of 1920 × 1080 are collected at 30 Hz using the smartphone camera during driving. The collected road-surface images are entered into a simple DNN model for the detection of road-surface anomalies, and images containing road-surface anomalies are identified and saved. As each image is captured, three-axis acceleration data are collected over a 3 s period at 100 Hz. The collected images and acceleration data are then immediately transmitted to a server, where they are stored. These data can then be viewed on a website that displays this information on a map within one minute, allowing the road-management entity to access up-to-date information describing road conditions.

Please note that the data acquisition method proposed in this study does not collect detailed road-surface anomaly information (such as cracking) at the same level that existing expensive equipment is able to; it only collects information describing the presence or absence of road-surface anomalies and the corresponding variation of acceleration. However, the proposed method uses low-cost, easily deployable technology to enable the rapid checking of dynamic information describing changes in the road condition a wide spatial and temporal range by using a plurality of data collection devices.

### 2.2. FCN-Based Road-Surface Anomaly Detection Model

**Figure 2:**
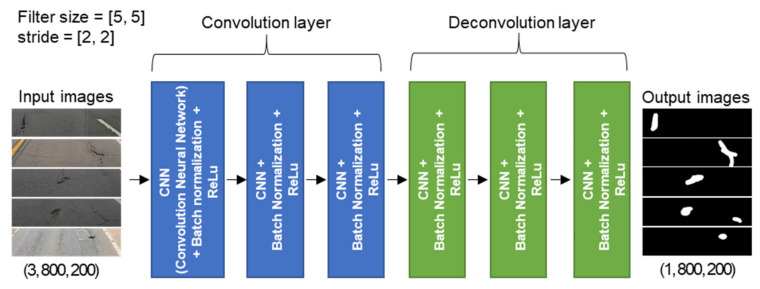
Structure of the FCN-based road-surface anomaly detection model.

Figure 2 shows the structure of the simple DNN model installed in the smartphone. This model was developed to classify road-surface anomalies by analyzing the images captured in real time while driving. The FCN was designed to have six layers, excluding the input layer, consisting of three stride convolution layers and three stride deconvolution layers. There was no pooling layer. In each convolution layer, a 5 × 5 filter traversed the entire image at 2 × 2 stride intervals for computation. A rectified linear unit (ReLU) was used as the activation function, and 1959 road-surface images were used as training and validation data. Of these images, 599 contained road anomalies with the remainder containing shadows, vehicles, road facilities, road markings, etc., to exclude environmental impacts. Images were not augmented, such as by adjusting the brightness to compensate for changes in the ambient lighting, so these tests were mainly conducted during daylight hours. The FCN used in this study had a structure similar to that developed in a previous study [28], but was simplified so that FCN-based road-surface anomaly detection and acceleration measurement could be simultaneously performed by a smartphone.

**Figure 3:**
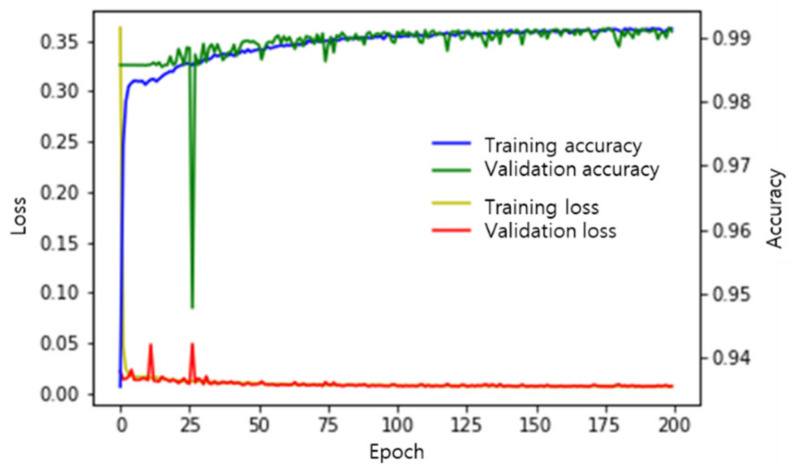
Loss and accuracy of the FCN inference model according to training epoch.

To train the FCN, the original 1920 × 800 images were cropped to 800 × 200. In each extracted image, the pixels associated with road-surface anomalies were labeled as 1 and the pixels without any anomaly were labeled as 0. (The output images in Figure 2 show the road-surface anomalies as pixel values of 255 to improve visibility, but their actual values were 1). Eighty percent of the images were used for model training, and the remaining 20% of the images were used for validation. The mean square error (MSE) was used as the loss function [29], and adaptive moment estimation (ADAM) was used as the optimization technique [30]. Figure 3 shows the loss and accuracy of the FCN inference model throughout the training iterations. The loss values for the training and validation data sets were measured in every epoch during training, and the model in which the loss value for the validation data set was the smallest was used.

### 2.3. Configuration of the Collected Data

Data were collected during driving on urban roads in Goyang City using the developed FCN-based road-surface anomaly detection and acceleration data collection system. As shown in Table 1, 1896 images and the corresponding three-axis acceleration data were acquired, many with various types of manually identified anomalies. Local anomalies, such as potholes, manholes, and traces of repairs to damaged road surfaces were observed in 241 of the acquired images. Lateral road-surface anomalies, such as speed bumps, bridge expansion joints, and lateral joints and cracks were confirmed in 195 images. Joints and cracks that were continuous in the longitudinal direction were found in 457 images, indicating that longitudinal anomalies accounted for the largest percentage of captured anomalies. The remaining 1003 images contained no road-surface anomalies because the FCN model was otherwise likely to misinterpret the shadows of trees, road signs, nearby vehicles, and insignificant cracks as road-surface anomalies. Indeed, the model’s performance in distinguishing road-surface anomalies was previously very low because sufficient training data were not available compared with the input data [28]. Accordingly, separate image argumentation was not performed. However, it was determined that an FCN model with low performance was suitable for the comparison and analysis of the measured results for various road-surface conditions because it was augmented by the acceleration data collected during the acquisition of both normal and abnormal road-surface images.

## 3. Preprocessing of Acquired Acceleration Data

### 3.1. Typical Images and Collected Acceleration Data

**Figure 4:**
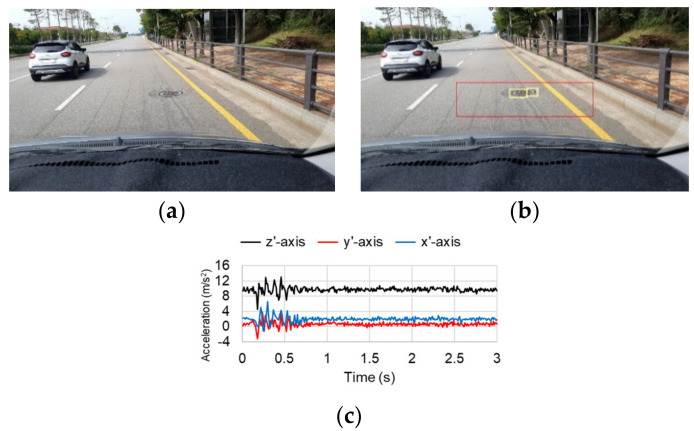
Typical image and accelerations obtained with a smartphone camera and three-axis accelerometer: (**a**) Original image; (**b**) Predicted anomaly; (**c**) Obtained accelerations.

Figure 4 shows a typical image and the corresponding acceleration data. The smartphone camera was attached to the upper part of the windshield of a traveling vehicle to capture and store images of the road surface, shown in Figure 4a. Eriksson et al. [7] installed accelerometers in three different places inside a vehicle (the dashboard, the right side of the windshield, and in the embedded PC) and compared the signal quality, finding that the accelerometers attached to the dashboard and windshield produced similarly high-quality signals. Thus, Eriksson et al. [7] selected the dashboard, where it was easiest to attach the sensor; in this study, the windshield was selected as the installation location because it was necessary to simultaneously photograph the road surface using the smartphone camera. The collected images were input into the FCN model to detect road-surface anomalies. The road-surface anomalies in the region-of-interest (ROI) marked with a red box in Figure 4b were detected and are marked with yellow boxes. When the road-surface anomalies were detected, the three-axis accelerations (*x*′, *y*′, *z*′) of the smartphone were measured for 3 s, as shown in Figure 4c. In Figure 4, a manhole was detected as an anomaly in the ROI, and the corresponding acceleration change was measured by the smartphone. It can be observed that the measured accelerations varied significantly between 0 and 1 s as the vehicle passed over the manhole.

**Figure 5:**
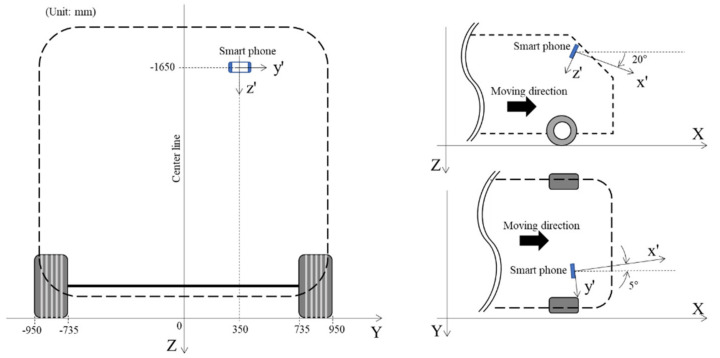
Installation of the smartphone to conduct road-surface anomaly detection.

As shown in Figure 4a,b, road-surface images were captured with the smartphone in a rotated position. A fixing device was used to maintain a constant posture of the smartphone during driving. Figure 5 shows the initial orientation of the smartphone, which was attached to the windshield of the test vehicle. These tests were conducted using a sport utility vehicle (SUV) that was 1.925 m high, 1.920 m wide, and 5.15 m long, with a front wheel tread of 1.685 m and 0.215 m wide tires. The smartphone was installed on the inner surface of the vehicle windshield at a height of approximately 1.65 m from the ground, and at a distance of 0.35 m toward the passenger seat from the center of the vehicle. The direction of gravity was defined as the *Z*-axis of the global coordinate system, and the *X*- and *Y*-axes were defined as the longitudinal and lateral directions of the vehicle, respectively. The orientation of the smartphone was determined by rotating it 20° around the *y*’-axis so that its camera could visualize the road surface in the *X*–*Z* plane. The smartphone was also rotated 5° around the z′-axis so that the camera could visualize the centerline of the road in the *X*–*Y* plane. The smartphone was installed so that it could not rotate around the *x*′-axis.

### 3.2. Conversion of Acceleration Measurements into the Global Coordinate System

As the local coordinate system of the smartphone changes continually during driving, the acceleration data must be converted to the corresponding values in the fixed global coordinate system so that the changes in acceleration during driving can be compared under the same conditions. Accordingly, the process of converting the acceleration measurements in the local coordinate axis system of the smartphone into the global coordinate system, in which the direction of gravity was defined as the *Z*-axis, is depicted in Figure 5.

The accelerations measured by the smartphone can be converted into accelerations in the global coordinate system using Euler angles and a rotation matrix that expresses the rotation of an object. The Euler angles are the three angles used to express the direction of an object in 3D space. In Figure 5, the Euler angles are denoted as ϕ for the *X*-axis, θ for the *Y*-axis, and ψ for the *Z*-axis. Assuming that the change in the rotation angle of two of the axes is zero (and given that the Euler angle of the remaining axis is excluded), the relationships between the accelerations measured in each of the axis directions of the smartphone and the accelerations converted to the global coordinate system can be expressed using Equations (1)–(3):

(1) $$\left[\begin{array}{ccc} a_{X} & a_{Y} & a_{Z} \end{array}\right]=\left[\begin{array}{ccc} a_{x’} & a_{y’} & a_{z’} \end{array}\right]\left[\begin{array}{ccc} 1 & 0 & 0\\ 0 & \cos\phi & -\sin\phi \\ 0 & \sin\phi & \cos\phi \end{array}\right]$$

(2) $$\left[\begin{array}{ccc} a_{X} & a_{Y} & a_{Z} \end{array}\right]=\left[\begin{array}{ccc} a_{x’} & a_{y’} & a_{z’} \end{array}\right]\left[\begin{array}{ccc} \cos\theta & 0 & \sin\theta \\ 0 & 1 & 0\\ -\sin\theta & 0 & \cos\theta \end{array}\right]$$

(3) $$\left[\begin{array}{ccc} a_{X} & a_{Y} & a_{Z} \end{array}\right]=\left[\begin{array}{ccc} a_{x’} & a_{y’} & a_{z’} \end{array}\right]\left[\begin{array}{ccc} \cos\psi & -\sin\psi & 0\\ \sin\psi & \cos\psi & 0\\ 0 & 0 & 1 \end{array}\right]$$

where $a_{X}$, $a_{Y}$, and $a_{Z}$ are the respective acceleration values based on the global coordinate system, and $a_{x’}$, $a_{y’}$, and $a_{z’}$ are the acceleration values measured by the smartphone.

In this study, the Euler angles were rotated clockwise along the order of the *XYZ* axes. Therefore, the values of $a_{X}$, $a_{Y}$, and $a_{Z}$ obtained using Equation (1) were substituted into Equation (2) as $a_{x’}$, $a_{y’}$, and $a_{z’}$. Likewise, the values of $a_{X}$, $a_{Y}$, and $a_{Z}$ obtained with Equation (2) were substituted into Equation (3) as $a_{x’}$, $a_{y’}$, and $a_{z’}$. Finally, the accelerations in the global coordinate system that reflect the rotation of all coordinates were calculated.

### 3.3. Estimation of the Vehicle Wheel Paths

In total, 1896 road-surface images and the corresponding acceleration data were obtained by the smartphone-based system. Among these, 893 images contained road-surface anomalies, as shown in Table 1. If the vehicle wheels do not pass over the road-surface anomalies in the observed images, it is difficult to distinguish their effects from the acceleration data corresponding to normal road images. Therefore, it is necessary to estimate whether the wheels will pass over the anomalies identified in the road-surface images.

**Figure 6:**
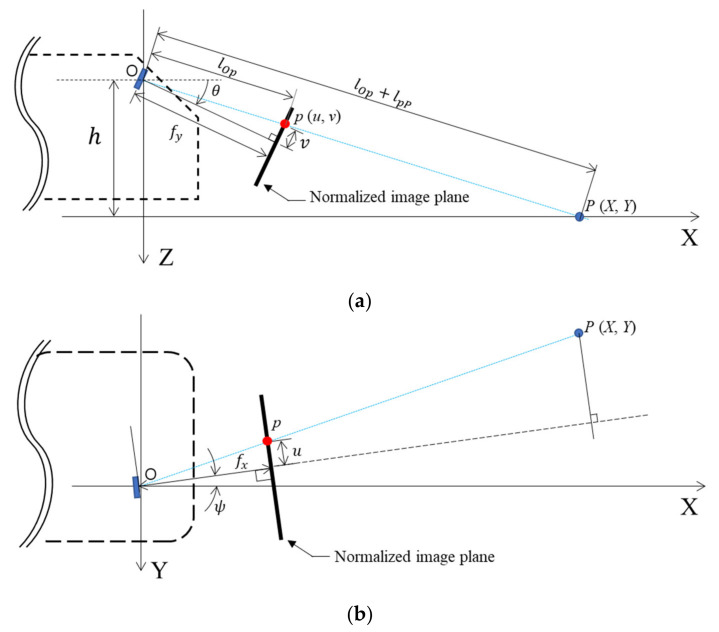
Geometric forms of the image plane (*u*, *v*) and ground plane (*X*, *Y*): (**a**) *XZ* plane; (**b**) *XY* plane.

To examine whether the wheels will pass over the anomalies detected in the road-surface images, it is necessary to calculate the coordinates of the anomalies when each pixel of the image is subjected to ground projection. Figure 6a shows a point *p* in the pixel coordinate system of a road-surface image and the corresponding point *P* in the ground coordinate system. The two points lie on a straight line. If the pixel coordinate system changes depending on the camera focal length (*f*), principal point (c), height (h), and rotation angle (ψ), the coordinates of point *p* in the pixel coordinate system—that is, (x, y)—will also change. Therefore, the coordinate system of point *p* was normalized to eliminate the inherent internal parameters of the camera, as shown in Equations (4) and (5):

(4) $$u=\left(x-c_{x}\right)/f_{x}$$

(5) $$v=\left(y-c_{y}\right)/f_{y}$$

where x and y are the coordinates of point *p* in the pixel coordinate system (the images used in this study had a resolution of 1920 × 1080, therefore x ranged from 0 to 1920 and y ranged from 0 to 1080); $c_{x}$ and $c_{y}$ are the coordinates of the principal point of the camera in the pixel coordinate system (assumed to be equal to 960 and 540, respectively, denoting the median values of *x*); and $f_{x}$ and $f_{y}$ denote the focal lengths of the smartphone camera telephoto lens, equal to 1415.06 and 795.97 pixels, respectively. When the x and y pixel values obtained from the image are substituted into Equations (4) and (5), the origin becomes the principal point and the focal length between the image plane and the camera origin is normalized to unity. The geometric relationship between the normalized pixel coordinates (u, v) and ground coordinates (X, Y) in Figure 6a,b can be used to obtain X and Y, which are respectively the longitudinal and lateral distances from the origin (where the camera is installed) to point *P*, as shown in Equations (6) and (7):

(6) $$X=h\cdot \tan\left(\frac{\pi }{2}+\theta -\tan^{-1}v\right)$$

(7) $$Y=u\cdot \frac{l_{Op}+l_{pP}}{l_{Op}}$$

where $l_{Op}$ is the distance from the principal point to point p in the *XZ* plane, defined to be equal to $\sqrt{1+v^{2}\;}$, and $l_{pP}$ is the distance from point p to point *P* in the *XZ* plane. Thus, $l_{Op}+l_{pP}$ is the distance from the principal point to point P, and is defined to be equal to $\sqrt{X^{2}+h^{2}\;}$. Considering the rotation angle (ψ) of the smartphone in the *z*’-axis direction in Figure 6b, Equations (6) and (7) can respectively be expressed in the forms of Equations (8) and (9):

(8) $$X=h\cdot \tan\left(\frac{\pi }{2}+\theta -\tan^{-1}v\;\right)\cdot \cos\psi$$

(9) $$Y=u\cdot \frac{l_{Op}+l_{pP}}{l_{Op}}-X\cdot \sin\psi$$

**Figure 7:**
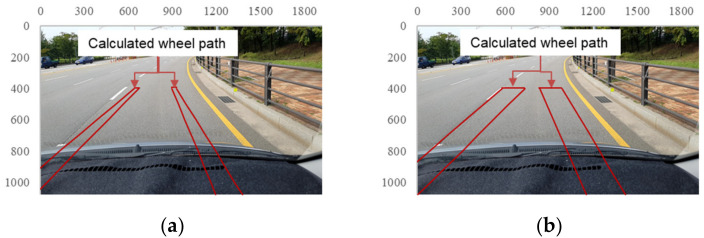
Areas associated with wheel paths on acquired images (red-highlighted areas): (**a**) ψ = 5°; (**b**) ψ = 2.5–7.5°.

Because the front wheel tread of the SUV used in these tests was 1.685 m and the tires were 0.215 m wide, the wheels were located from −0.95 to −0.735 m and from 0.735 to 0.95 m from the longitudinal axis of the vehicle, as shown in Figure 5. In addition, because the camera was located 0.35 m from the longitudinal axis toward the passenger seat, the *Y*-axis coordinates of the wheels were located from −1.3 to −1.085 m and from 0.385 to 0.6 m in the global coordinate system. In Equations (8) and (9), h, θ, and ψ, which are related to the orientation of the smartphone camera, and Y, which describes the location of the wheels, all have known values. Thus, the normalized pixel coordinates (u, v) can be calculated when a value is entered for X by assuming that the vehicle is traveling in a straight line. Moreover, the pixel coordinates (x, y) can be calculated using (u,v) and Equations (4) and (5). Figure 7a shows a road-surface image in which the calculated pixel coordinate values for the wheel paths on the road surface are indicated in red. The range of ψ was then adjusted from 2.5° to 7.5° owing to the curved trajectory of the vehicle during its motion and the slight rotation of the camera. Based on these values, the areas corresponding to the wheel paths were determined as shown in Figure 7b.

When road anomalies were detected using the FCN model, the pixel coordinates of the detected areas were stored. Then, the cases in which the pixel coordinates of the road anomalies detected by the FCN model overlapped the wheel paths, shown in the image in Figure 7b, were identified. Among the 893 road-surface images containing road-surface anomalies, 293 showed that the pixel coordinates of the road anomalies overlapped the wheel paths.

## 4. Acceleration Data Acquisition Results and Analysis

### 4.1. Histogram of the Maximum Variation of Z-Axis Acceleration According to the Time Needed to Acquire Acceleration Data

**Figure 8:**
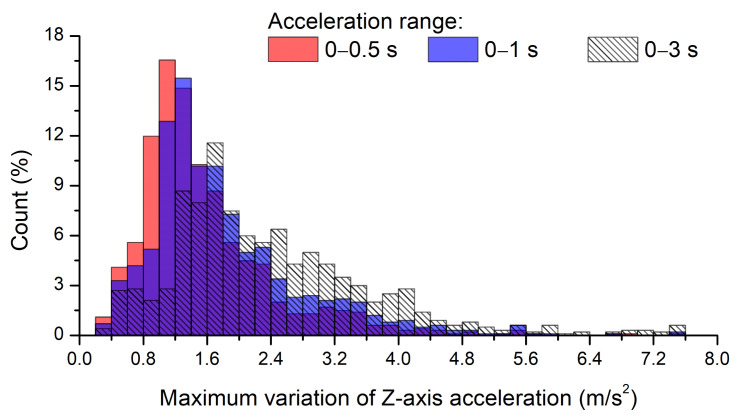
Histogram for the maximum variation of *Z*-axis acceleration during different data acquisition periods.

In this study, acceleration data were acquired for 3 s at 100 Hz, but it was necessary to determine whether all the acquired acceleration data were needed or only the acceleration signals for the ROI in the images. Figure 8 shows the histogram for the maximum variation of *Z*-axis acceleration in 1003 images without road-surface anomalies within the selected ROI. The maximum variation of *Z*-axis acceleration represents the maximum difference between the *Z*-axis acceleration acquired in a certain time range (for detection) and gravitational acceleration. Thus, the maximum variations of the *Z*-axis acceleration data collected within time ranges of 0–3, 0–1, and 0–0.5 s were found to exhibit average values of 2.436, 1.834, and 1.582 m/s^2^, respectively, and median values of 2.152, 1.548, and 1.354 m/s^2^, respectively. Clearly, the maximum acceleration variation tended to decrease as the acceleration acquisition range decreased. In addition, paired t-tests with a significance level of 0.05 were performed between accelerations collected during the 0–3 and 0–1 s time ranges and the 0–3 and 0–0.5 s time ranges; the t-values were determined to be 19.24 and 24.43, respectively, confirming that mean values were significantly different, as the critical value of 1.96 was exceeded.

During the test, the vehicle was driven at an average speed of 54.9 km/h (=15.26 m/s). Thus, distances equal to 45.78, 15.26, and 7.63 m were covered in the 0–3, 0–1, and 0–0.5 s time ranges, respectively. The y values of the top and bottom of the ROI analyzed by the FCN model in the pixel coordinate system were 450 and 650, respectively. The *X* values of the top and bottom parts of the ROI calculated using Equations (5), (6) and (8) were equal to 6.38 and 3.09 m, respectively. Therefore, the acceleration signals collected in the 0–3 or 0–1 s ranges included data generated after the vehicle passed the selected ROI. Although there were no road anomalies in the ROI in 1003 road-surface images, the variation of acceleration owing to road anomalies after the ROI were included. Therefore, in this study, only the acceleration data in the range of 0–0.5 s was used for the analysis of the results for road-surface anomalies.

### 4.2. Analysis of the Histogram for the Variation of Z-Axis Acceleration

**Figure 9:**
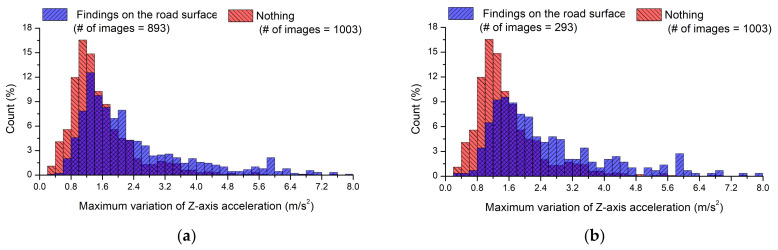

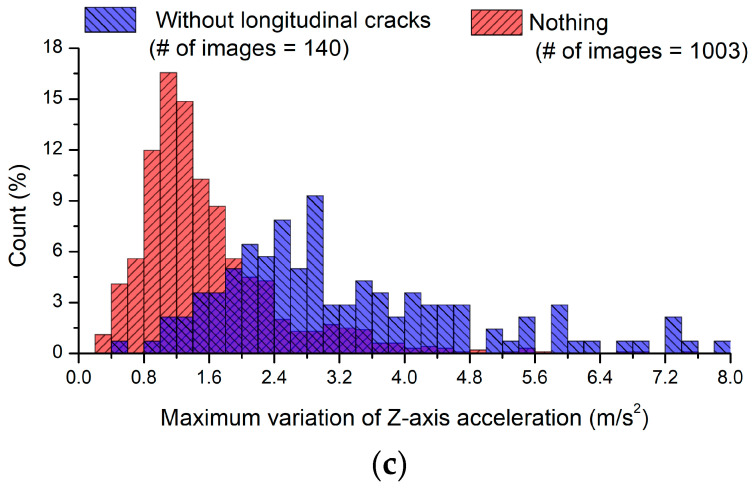
Histograms as a function of the maximum variation of *Z*-axis acceleration with and without detected cracks in acquired images: (**a**) Results depending on the presence of road anomalies; (**b**) Classification based on wheel paths; (**c**) Results obtained by excluding longitudinal cracks.

The vehicle acceleration in the direction of gravity (*Z*-direction) was mainly used to identify road anomalies in this study. This section presents the preprocessing applied to the smartphone acceleration data to obtain better results. Figure 9 shows the histograms for the maximum variation of *Z*-axis acceleration obtained corresponding to each image. Figure 9a shows a comparison of acceleration signals with and without road anomalies in the ROI after the acquired images were re-examined by the FCN model. The results without anomalies in the ROI are clearly the same as those in the range of 0–0.5 s in Figure 8. When there were road anomalies in the ROI, the average variation of *Z*-axis acceleration was 2.46 m/s^2^, and the median variation of *Z*-axis acceleration was 1.92 m/s^2^. It was difficult to distinguish between the maximum variation of *Z*-axis acceleration according to the presence or absence of road anomalies because similar ranges were observed. The distinction was particularly difficult to observe when the maximum variation of *Z*-axis acceleration ranged from 1.0 to 2.0 m/s^2^. Furthermore, it appears that distinguishing between the presence and absence of road anomalies in the ROI could be even more difficult using accelerations collected over the 0–1 s or 0–3 s periods.

Figure 9b shows the results obtained by excluding the road-surface images in which the pixel coordinates of the road anomalies did not overlap with the wheel paths. When the wheel path was considered, the average maximum variation of *Z*-axis acceleration was 2.66 m/s^2^ and the median was 2.10 m/s^2^. Thus, normal conditions were more accurately distinguished from conditions with road-surface anomalies when accounting for the wheel paths than when using all images with road-surface anomalies.

Table 1 indicates that images with longitudinal anomalies represented 51% of all images with road anomalies. Longitudinal anomalies; however, cause smaller variations in acceleration than lateral or local anomalies, similar to those in cases without road-surface anomalies. Thus, in Figure 9c, acceleration data associated with longitudinal anomalies were excluded from the 293 images in which the pixel coordinates of road anomalies overlapped with the wheel paths. The average variation of *Z*-axis acceleration was 3.28 m/s^2^ and the median acceleration was 2.72 m/s^2^. These results indicate notable differences from the cases without road-surface anomalies. For the three pairs of data shown in Figure 9, *t*-tests were performed with a significance level of 0.05. The t-values in Figure 9a–c were calculated to be 14.46, 10.44, and 10.36, respectively, indicating that there were statistically significant differences in mean values as the thresholds of 1.961, 1.967, and 1.976 were exceeded.

### 4.3. Histogram Analysis for the Ratio of Y- to Z-Axis Accelerations

**Figure 10:**
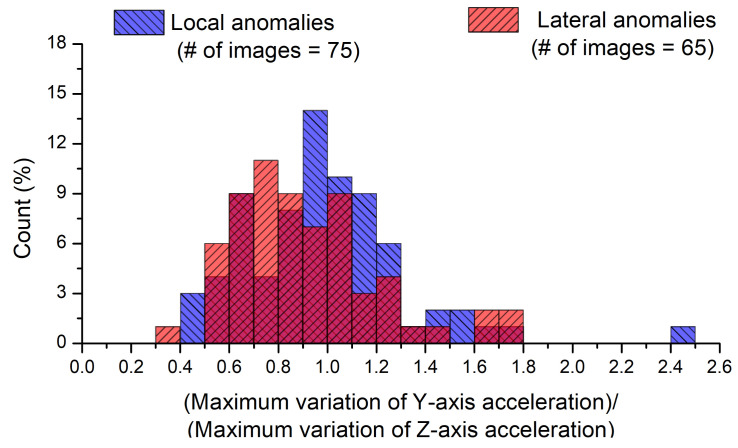
Histograms for the ratio of the maximum variation of *Y*-axis acceleration to that of *Z*-axis acceleration.

The ratio of the *Y*- to *Z*-axis accelerations was used as a criterion to distinguish symmetrical road-surface anomalies (such as lateral anomalies) from asymmetrical road-surface anomalies (such as local anomalies) by identifying the rolling of the vehicle [7]. The *Y*-axis acceleration may vary significantly when the vehicle passes over asymmetrical anomalies, but its change may be smaller for symmetrical anomalies. Figure 10 shows the histograms for the ratio of the maximum variation of the *Y*-axis acceleration to that of the *Z*-axis acceleration obtained during the driving test according to local and lateral anomalies. The acceleration data obtained from 140 road-surface images were used after the exclusion of those in which the wheels were expected to pass over a road-surface anomaly, longitudinal joint, or crack with only a small acceleration change. The maximum variation of *Z*-axis acceleration represents the maximum acceleration change from gravitational acceleration (equal to 9.81 m/s^2^) in the range of 0–0.5 s as aforementioned, whereas the maximum variation of the *Y*-axis acceleration represents the maximum acceleration change from 0 m/s^2^ in the range of 0–0.5 s. Figure 10 shows that the histogram obtained from the local anomalies is shifted to the right compared to the histogram obtained from the lateral anomalies, thus indicating a higher maximum acceleration variation ratio. An average value of 0.99 was observed for local anomalies and 0.81 for lateral anomalies. The histograms for the two conditions; however, were both contained within similar ranges between 0 and 2, and there was no significant difference between their distributions. Moreover, a *t*-test was conducted with a significance level of 0.05 for the two data sets shown in Figure 10. The calculated *t*-value was 0.25, and the threshold was 0.80; therefore, the difference between the mean values was not statistically significant. Several cases existed in which lateral anomalies were not symmetrical because they were obliquely situated. Even if lateral anomalies remain symmetrical, the *Y*-axis acceleration may vary significantly because the wheels on each side reach the lateral anomaly at different times depending on the direction of the moving vehicle, and the spatial orientation of the lateral anomaly.

## 5. Image and Acceleration Results and Discussion

**Figure 11:**
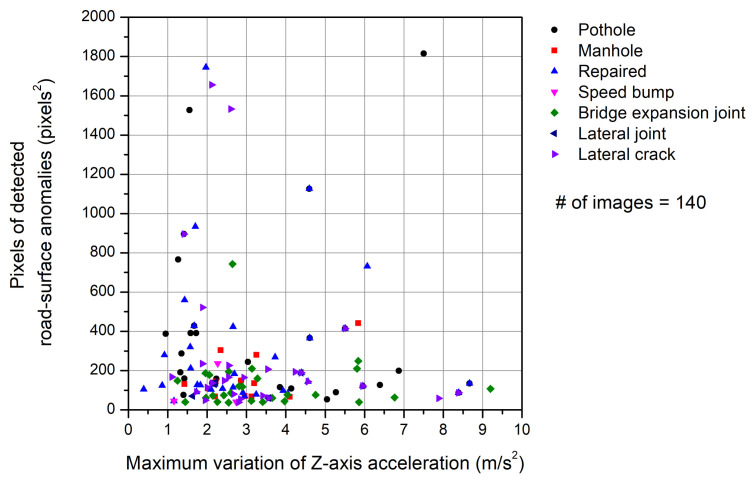
Relationship between the pixel area of the road anomalies detected by the FCN model and the maximum variation of *Z*-axis acceleration.

**Figure 12:**
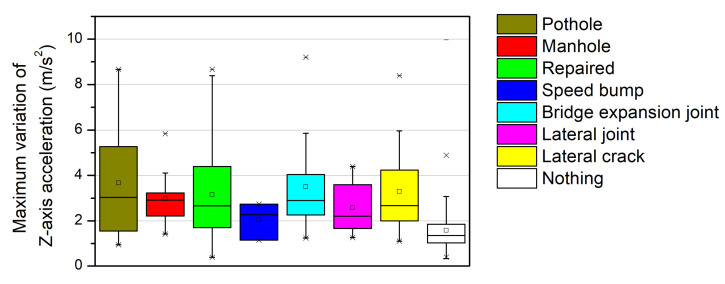
Box plots of the maximum variation of *Z*-axis acceleration for different road-surface anomalies.

**Figure 13:**
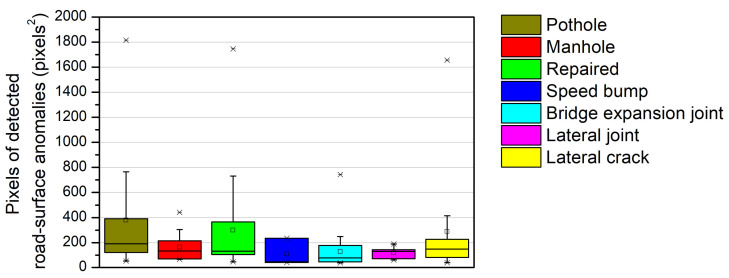
Box plots of the pixels of detected road-surface anomalies for different road-surface anomalies.

Figure 11 shows the relationship between the area (in pixels^2^) of the road-surface anomalies and the maximum variation of *Z*-axis acceleration. Only the results for the 140 road-surface images defined in Section 4.3 are shown. In general, the change in the *Z*-axis acceleration is expected to be larger as the size of the road-surface anomaly increases. It is difficult; however, to find any correlation between the two parameters in Figure 11. To identify the reason for the irrational relationship between the pixel size of the detected road-surface anomalies and the maximum variation of *Z*-axis acceleration, box plots are drawn for each parameter as shown in Figure 12 and Figure 13.

Figure 12 shows box plots for the maximum variation of *Z*-axis acceleration for different types of road anomalies. In the case of the nothing on the road surface, most of the maximum variations of the *Z*-axis accelerations were less than 2 m/s^2^. However, when anomalies were detected on the road surface, the variations of the *Z*-axis accelerations were mostly >2 m/s^2^. The three box plots on the left of Figure 12 show the results for three types of local anomalies. In the cases of potholes and repaired road surfaces, which have irregular shapes, the variations in acceleration were more extensively distributed than in the other cases. Meanwhile, for manholes, which have a constant geometry (e.g., circular), the change in value was concentrated within a narrow range. Additionally, box plots for the four different types of lateral anomalies can be observed on the right in Figure 12. The variation of *Z*-axis acceleration for the speed bump was the smallest owing to the deceleration just before passing, though the values for the remaining three types of lateral anomalies yielded similar distributions.

Figure 13 shows box plots for the pixels of detected road-surface anomalies. Among the local anomalies, the pothole and the repaired road surface exhibited the widest distribution of pixels and the manhole exhibited the narrowest distribution (similar to the variations of *Z*-axis acceleration shown in Figure 12). However, the maximum variations of *Z*-axis acceleration of the lateral anomalies are similar to those of the repaired shape in Figure 12, but their pixel distributions are smaller than that of the repaired shape in Figure 13. The detected pixel values in the case of the lateral anomalies were smaller than those in the case of the local anomalies given that pixels can be distributed as a narrow continuous line in the lateral direction, but may still cause a significant acceleration change. Therefore, it is necessary to accurately estimate the geometry of these anomalies, as different instances of road-surface damage with the same area may cause different changes in acceleration depending on the depth of the damage. The FCN model used in this study can provide approximate information describing the presence of road-surface anomalies and their locations, but not their depths. Therefore, a more precise model is required to represent severity based on the detected pixel area of the road-surface anomalies in Figure 11.

**Figure 14:**
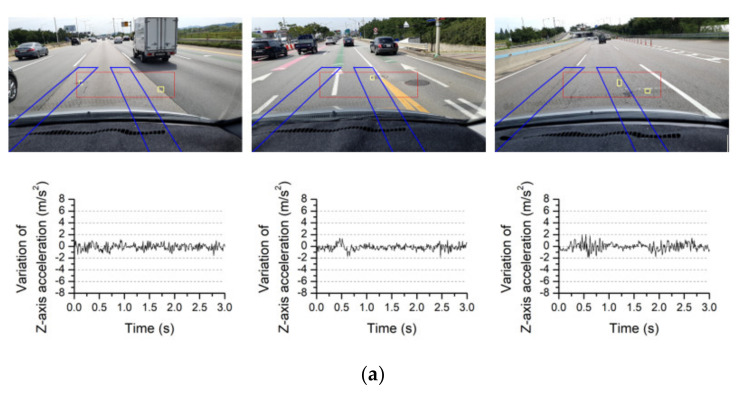

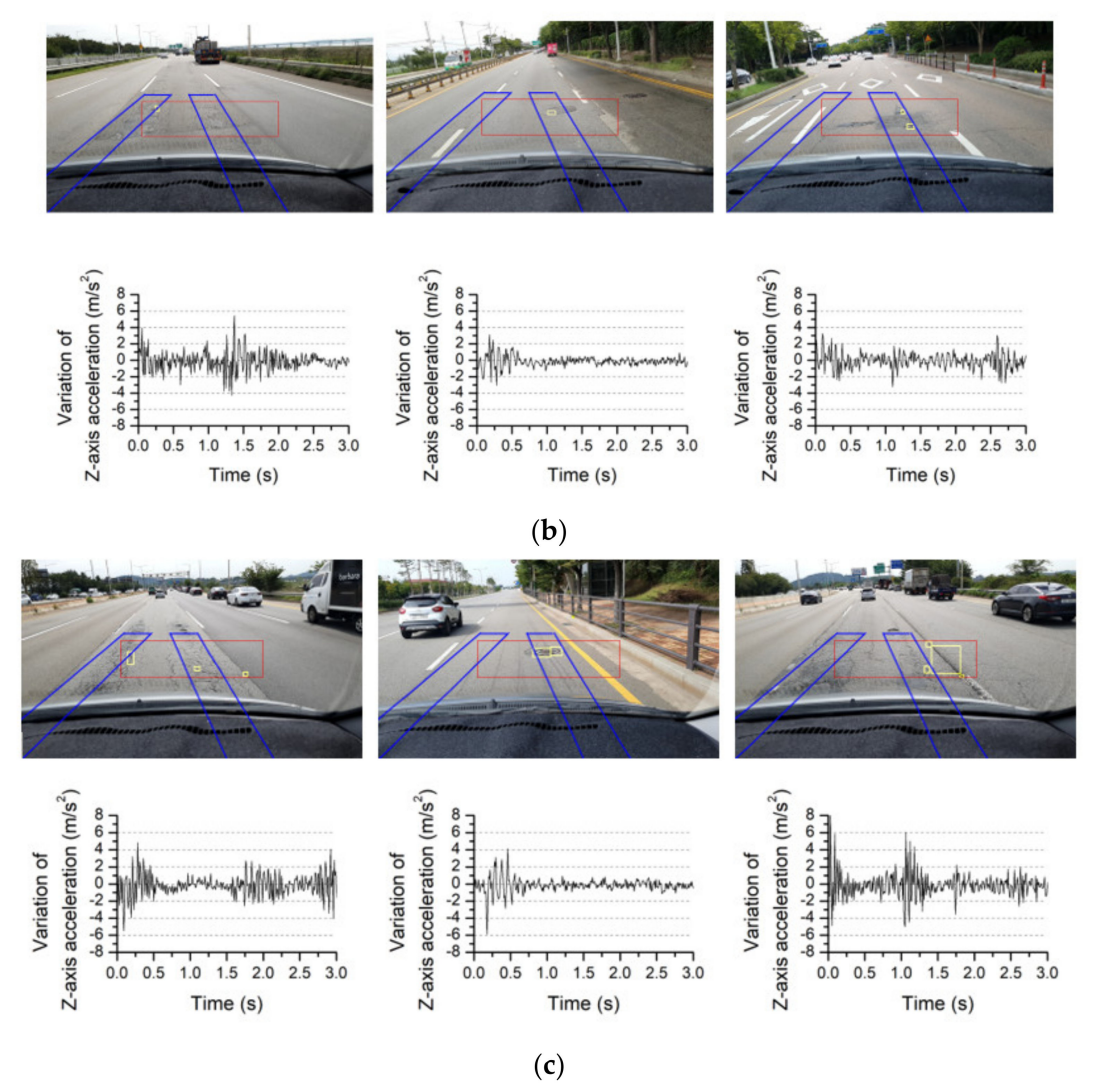
Typical road-surface images and corresponding variations of *Z*-axis acceleration for different quartiles of maximum variations of *Z*-axis accelerations: (**a**) Maximum variation of *Z*-axis acceleration < 2 m/s^2^; (**b**) Maximum variation of *Z*-axis acceleration ≥ 2 m/s^2^ and < 4 m/s^2^; (**c**) Maximum variation of *Z*-axis acceleration ≥ 4 m/s^2^.

The acceleration data corresponding to the 140 images defined in Section 4.3 were classified according to the range of the maximum variation of *Z*-axis acceleration using quartiles of the data. The values of the first quartile (Q1 = 1.90 m/s^2^) and third quartile (Q3 = 4.2 m/s^2^) of the *Z*-direction maximum acceleration data were obtained and simplified to near-integer values. When the maximum variation of *Z*-axis acceleration was less than 2 m/s^2^, as shown in Figure 14a, values similar to the acceleration data in normal conditions were observed. Notably, cases in which small changes in acceleration appear to be caused by road anomalies located at the edges of the wheel paths were included in this case, as shown in the second image of Figure 14a. Figure 14b shows the road-surface images for which the maximum variation of *Z*-axis acceleration was between 2 and 4 m/s^2^. Anomalies that may affect the wheel paths can be detected, including a manhole and local anomalies, such as small potholes. Images in which the road-surface condition was generally uneven were also included in this case. Figure 14c shows the images for which the maximum variation of *Z*-axis acceleration was 4 m/s^2^ or greater. Anomalies that cause severe changes in acceleration and may require repairs can be frequently found in the driving path. In addition, changes in acceleration of 4 m/s^2^ or greater may occur when a manhole with a large height difference is passed or when the repaired road surface is uneven.

The severity of road-surface anomalies can be identified by comparing the images with the maximum variation of *Z*-axis acceleration, as shown in Figure 14. Therefore, when it is difficult to quantitatively identify the severity of a road-surface anomaly by estimating its area and depth from the road-surface images using the FCN model, it is possible to do so by converting the acquired three-axis accelerations into accelerations relative to the gravity axis, and classifying them into ranges at the moment of wheel passage. If an FCN model that can more accurately distinguish images is prepared and more driving data are accumulated in various conditions in the future, it will be possible to provide more detailed identification of road-surface anomalies by combining images and acceleration data.

## 6. Conclusions

In this study, a system was developed to identify road-surface anomalies in collected images using an FCN model while simultaneously processing three-axis accelerations collected during the concurrent period of time. The developed system was installed on a smartphone that was placed on a vehicle windshield and tested on public roads. The proposed system, which combined an FCN-based road-surface anomaly detection method with accelerometer-based data acquisition, allowed the severity of FCN-identified road-surface anomalies to be determined by classifying the concurrent variations in gravitational-axis accelerations into certain ranges. Existing systems that use special inspection vehicles to identify road-surface anomalies are expensive to operate on a large scale; however, the proposed system incurs significantly lower costs and can be extensively distributed via widely owned and readily available smartphones. The system demonstrated in this study is thus promising for the widespread application of automatic road-surface anomaly detection. However, the present study was unable to determine the effect of vehicle type and vehicle speed on the clarity of the obtained information, which should be a target of future research to improve the accuracy of the proposed system.

## Figures and Tables

**Table 1:** Classification of anomalies in road-surface images acquired by the smartphone camera.

Findings on the Road Surface									Nothing Detected on the Road Surface	Total
Local Anomalies			Continuous Anomalies							
			Lateral Anomalies				Longitudinal Anomalies			
241			195				457			
Pothole	Manhole	Repaired	Speed Bump	Bridge Expansion Joint	Lateral Joint	Lateral Crack	Longitudinal Joint	Longitudinal Crack		
50	60	131	9	44	42	100	113	344	1003	1896

## Data Availability

Not applicable.
